# Pain-related and psychological factors mediate the effect of personality on health-related quality of life. A study in breast cancer survivors with persistent pain

**DOI:** 10.3389/fpsyg.2023.1063920

**Published:** 2023-07-07

**Authors:** Tommi Aho, Hanna Harno, Jari Lipsanen, Eija Kalso, Reetta Sipilä

**Affiliations:** ^1^Department of Anaesthesiology, Intensive Care and Pain Medicine, University of Helsinki and Helsinki University Hospital, Helsinki, Finland; ^2^Sleep Well Research Programme, University of Helsinki, Helsinki, Finland; ^3^Clinical Neurosciences, Neurology, University of Helsinki and Helsinki University Hospital, Helsinki, Finland; ^4^Department of Psychology and Logopedics, Faculty of Medicine, University of Helsinki, Helsinki, Finland; ^5^Department of Pharmacology, University of Helsinki, Helsinki, Finland

**Keywords:** persistent pain, personality, breast cancer, health-related quality of life, temperament and character

## Abstract

**Introduction:**

Personality characteristics affect the long-term well-being and health-related quality of life (HrQoL) of breast cancer (BC) survivors. Persistent pain significantly affects psychosocial well-being and HrQoL in this patient group. We studied the effects of temperament and character via pain-related and psychological factors on dimensions of HrQoL in BC survivors.

**Methods:**

We studied 273 patients who had been treated for BC and who reported persistent pain at any site of the body in Brief Pain Inventory. The patients were recruited from a longitudinal cohort of patients 4–9 years after surgery for BC. Short-Form-36 inventory was used to assess physical and mental dimensions of HrQoL and Temperament and Character Inventory to assess dimensions of temperament and character. We used parallel mediation modeling for studying effects of temperament and character on physical and mental HrQoL.

**Results:**

A significant total effect was found for harm avoidance (HA) temperament (β_total_ = −0.665, *p* < 0.001) and character dimensions self-directedness (SD) (β_total_ = 0.609, *p* = 0.001) and cooperativeness (CO) (β_total_ = 0.584, *p* = 0.028) on physical and mental HrQoL. Additionally, different combinations of pain-related and psychological variables fully mediated the indirect effects of HA, SD, and CO on physical and mental HrQoL.

**Discussion:**

HA temperament is a potential emotional vulnerability factor for psychological burden and impaired HrQoL in BC survivors. Character dimensions SD and CO may protect from the negative effect of mood on HrQoL. The results provide new insights about the risk-and target-factors for clinical interventions and effective pain management to improve psychosocial well-being and HrQoL in BC survivors.

## Introduction

Breast cancer (BC) is the most common cancer among women in Western countries (Ferlay et al., 2021). Since overall BC survival has improved over time, health-related quality of life (HrQoL) has become an important topic to study and verify patient experience of health, functioning, and psychological adjustment after BC treatments. Long-term distress, fatigue, and persistent pain are underestimated outcomes in this patient group and known to associate with impaired HrQoL (Mokhatri-Hesari and Montazeri, 2020). Personality characteristics affect the course of long-term well-being of BC survivors (Durá-Ferrandis et al., 2017) and may additionally provide a target for clinical interventions to improve psychological well-being and HrQoL (Abrahams et al., 2018; Ye et al., 2018).

Persistent pain following BC treatments is often multifactorial (Andersen and Kehlet, 2011) and affects 13–30% of BC survivors (Meretoja et al., 2014; Wang et al., 2018) with various negative effects on patients’ psychological well-being, sleep (Mustonen et al., 2019) and HrQoL (Caffo et al., 2003; Mokhatri-Hesari and Montazeri, 2020). In this patient group, the psychosocial aspects of disability, such as mood and catastrophic thinking are primary targets for psychological interventions (Edwards et al., 2016; Abrahams et al., 2018). Personality, its vulnerability and protective factors, explain some of the vicious cycle of chronic pain-related disability (Edwards et al., 2016; Naylor et al., 2017).

Perceived health and experience of well-being are shaped by temperament and character according to the psychobiological model of personality (Cloninger et al., 1993, 2010). Temperament dimensions *novelty seeking* (NS), *harm avoidance* (HA), *reward-dependence* (RD), and *persistence* (P) refer to stable and moderately heritable mechanisms of behavioral activation, inhibition, and maintenance. Character dimensions *self-directedness* (SD), *cooperativeness* (CO), and *self-transcendence* refer to concepts of coping and maturation, psychosocial adaptability, and spirituality that have been learned during a lifespan (Cloninger et al., 2010).

A combination of higher levels of HA temperament and lower levels of character dimension SD has previously been associated with impaired HrQoL in BC survivors (Bonacchi et al., 2012; Laroche et al., 2017) but also shown to be prevalent in patients suffering from persistent pain (Conrad et al., 2013; Mokhatri-Hesari and Montazeri, 2020). Individual tendency for caution, fearfulness, and fatigability (high HA) combined with immature coping strategies and lacking an internal locus of control (low SD) (Cloninger et al., 1993) increase the risk for poor adaptation, illbeing, and psychopathology (Cloninger et al., 2010).

The dynamic balance between emotional vulnerability and protective coping abilities appears to be essential for promoting well-being during chronic disability (Cloninger et al., 2010; Edwards et al., 2016; Abrahams et al., 2018). Despite of growing evidence, the effects of personality on different dimensions of HrQoL are poorly understood (Huang et al., 2017). We aimed to study effects of temperament and character on physical and mental dimensions of HrQoL in BC treated patients with persistent pain. We hypothesized that pain-related and psychological factors indirectly convey the effect of temperament and character on HrQoL. Parallel mediation modeling was used as a statistical method. We hypothesized that the protective effect of psychological adaptability-related character would associate with better HrQoL.

## Materials and methods

### Patients and demographics

**Figure 1:**
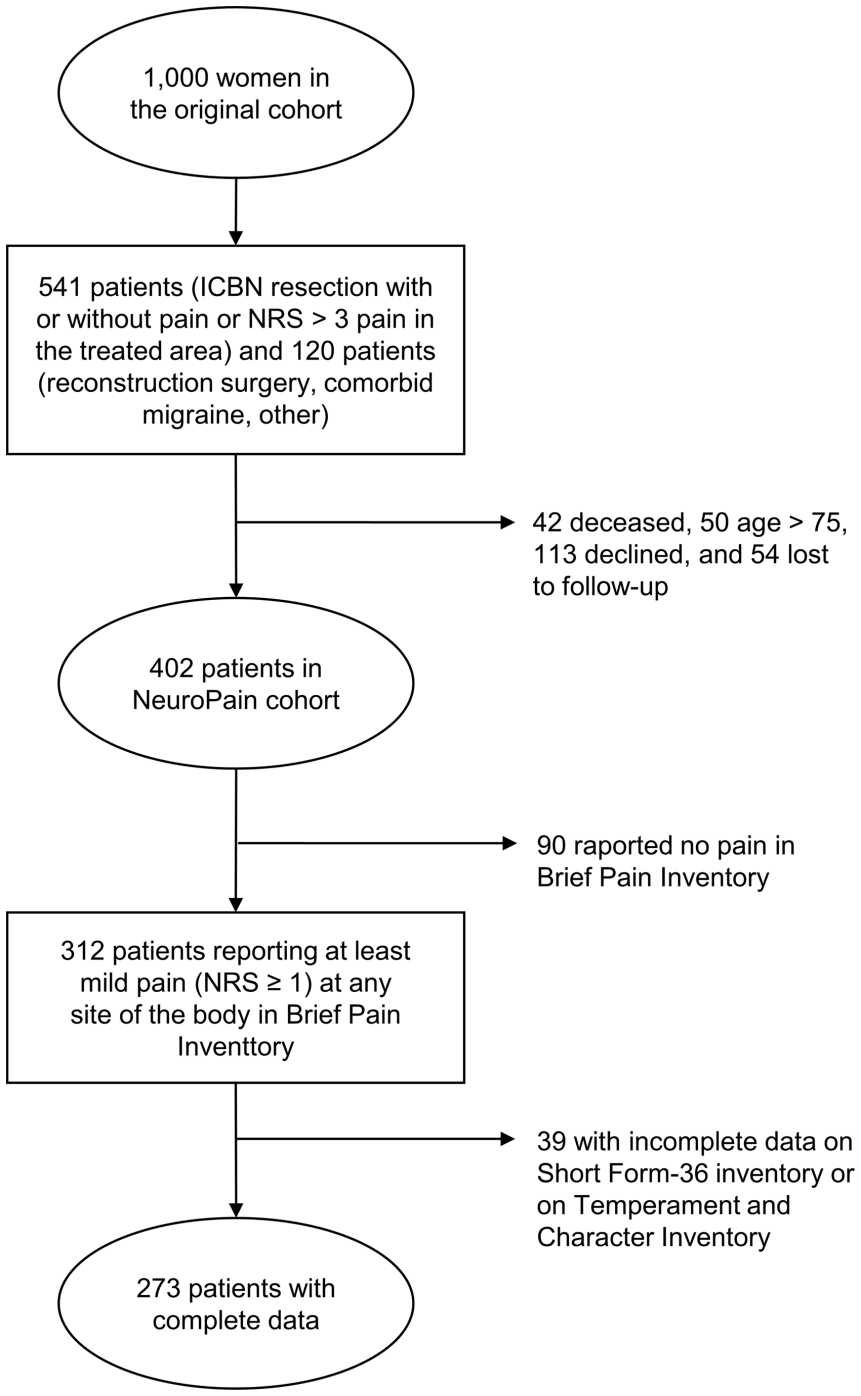
Flow chart of the patient recruitment. ICBN, intercostobrachial nerve; NRS, numerical rating scale.

We included patients from a subgroup of 402 women who had a research visit during 2014–2016 regarding perioperative surgical nerve injury and persistent post-surgical neuropathic pain (PPSNP) (Mustonen et al., 2019). That cohort was originally recruited from a previous longitudinal cohort of 1,000 women operated on for unilateral BC at the Helsinki University Hospital during years 2006–2010 (Kaunisto et al., 2013). The data was collected 4–9 years (mean 6.4 years) after the surgery. The patient flow of the original and sub cohort used in this paper have been reported in detail elsewhere (Kaunisto et al., 2013; Mustonen et al., 2019). Patients with active cancer treatments or metastatic cancer were excluded. Detailed description of patient recruitment is described in Figure 1. The study protocol was approved by the Ethics Committee of the Helsinki and Uusimaa Hospital District (reference number: 149/13/03/00/14) and registered in ClinicalTrials.gov (NCT 02487524). All patients provided a written informed consent. Data concerning previous surgery and BC treatments were extracted from patient records.

Patients who reported at least mild persistent pain (pain severity ≥ 1/10 on a Numerical Rating Scale (NRS), where 0 indicates no pain and 10 worst pain imaginable, at any site of the body (*N* = 312), were considered. In the second step, only patients who had a complete dataset on an independent variable (i.e., dimensions from a Temperament and Character Inventory) and on the main outcome variables (i.e., physical and mental HrQoL dimensions from a Short-Form-36 Health Survey) were included. The final study cohort consisted of 273 patients (Figure 1).

### Health-related quality of life

HrQoL was assessed by using the Finnish translation of the Short-Form-36 Health Survey (SF-36) questionnaire (Aalto et al., 1999), which has previously been used in BC survivors (Treanor and Donnelly, 2015). The SF-36 inventory includes 36 items with a scale from 0 to 100 which are organized into eight subscales: physical functioning, role-physical, bodily painlessness, general health, mental health, role-emotional, social functioning, and vitality.

For SF-36 dimensions of physical and mental HrQoL were formed as composite scores of the first four subscales (physical functioning, role-physical, bodily painlessness, and general health) and the last four subscales (mental health, role-emotional, social functioning, and vitality), respectively. Both dimensions ranged from 0 to 100 and higher scores indicate better performance in the dimension.

### Temperament and character

We used the Finnish translation of the 240-item self-administered Temperament and Character Inventory (TCI) (Miettunen et al., 2004) with a true-false scale to assess four temperament dimensions (*novelty seeking*, NS (range 0–40) (Cronbach α = 0.82); *harm avoidance*, HA (range 0–35) (Cronbach α = 0.89); *reward dependence*, RD (range 0–24) (Cronbach α = 0.71); and *persistence*, P (range 0–8) (Cronbach α = 0.56) and three character dimensions (*self-directedness*, SD (range 0–44) (Cronbach α = 0.79); *cooperativeness*, CO (range 0–42) (Cronbach α = 0.79); and *self-transcendence*, ST (range 0–33) (Cronbach α = 0.86), based on a psychobiological model of temperament and character (Cloninger et al., 1993).

NS describes the activation or initiation of behaviors in response to novelty, HA the inhibition or cessation of behaviors, RD the maintenance or continuation of ongoing behaviors, and P perseverance of behavior despite of frustration and fatigue (Cloninger et al., 1993). SD refers to self-determination, willpower, and ability to individual control, CO to social tolerance and empathy, and ST to spirituality (Cloninger et al., 1993). For all dimensions, higher scores indicate stronger tendency for dimension specific behavioral patterns.

### Psychological questionnaires and symptoms of insomnia

Hospital Anxiety and Depression Scale (HADS) was used to assess both anxiety (Cronbach α = 0.82) and depressive (Cronbach α = 0.88) symptoms (Bjelland et al., 2002). Pain Catastrophizing Scale (PCS) (Cronbach α = 0.93) was used to assess self-reported pain-related catastrophic thinking (Sullivan et al., 1995). Insomnia Severity Index (ISI) (Cronbach α = 0.91) was used for assessing self-reported symptoms of insomnia (Morin et al., 2011).

### Assessment of pain severity and pain interference

We used Brief Pain Inventory (BPI) to assess severity and interference of self-reported pains during the past week (Cleeland and Ryan, 1994). Patients reported pain severity and interference separately for the pains in the previously operated area (the breast, the axilla, the upper arm) and for the pains in any other site of the body (e.g., back pain, joint pain etc.). A pain severity variable was formed by calculating the mean NRS of the four items (i.e., the worst, the average, and the mildest pain during the week and pain at the moment). A pain interference variable was formed by calculating the mean NRS of the seven items assessing pain interference for different daily activities (i.e., general activity, walking, work, mood, enjoyment of life, relations with others, and sleep). Additionally, NRS was used for assessing pain severity during the clinical sensory examination included to the study protocol. We considered NRS ≥ 4/10 as moderate to severe pain (Gerbershagen et al., 2011).

### Statistical methods

Statistical analyses were performed using SPSS 25.0 for Windows (SPSS Inc., Chicago, IL, United States). Descriptive statistics are presented as mean (standard deviation, S.D.), median (interquartile range, IQR), or number (percentage). Pearson’s correlation was used to assess associations between the continuous variables. Results with *p* ≤ 0.05 were considered statistically significant.

For all statistical analyses, included variables were standardized by using the mean and S.D. of each variable [i.e., x-mean(x)/S.D.(X)]. Cronbach’s alpha (α) was used for reliability assessment for all psychological variables. For an exploratory statistical approach, we used parallel mediation modeling with multiple mediators. Mediation analyses were done by running a separate mediation analysis for each independent and dependent variable. The personality dimensions and the mediators were selected based on significant correlations between the independent variables and the main outcome variables to fulfill the criteria for mediation analysis (Preacher and Hayes, 2004). The other variables were used as a covariate in the models.

The parallel mediation analyses were performed by using the PROCESS add-on v.s. 16.1 in SPSS by using *model 4* (Hayes, 2013). Non-parametric bootstrapping (Preacher and Hayes, 2004) with 5,000 bootstrap samples were deployed to test the parallel mediational model of the elected mediators of the relationship between the temperament and character dimensions and the physical and mental HrQoL.

The entire effect of an independent variable on the outcome variable (total effect) and the effect of exposure of an independent variable on the outcome variable with (indirect effect, IE) and without (direct effect) the mediators were reported. The lower limit (LL) and the upper limit (UL) of the confidence interval (CI) were used to test statistical significance of the IE (Preacher and Hayes, 2004).

## Results

### Patient characteristics

Demographics, surgery, and treatment related factors of the 273 included patients are presented in Table 1. The mean age of the included patients was 61.7 years (range from 39 to 75 years). Most of the patients had intraductal carcinoma (64.5%) and they had had breast conserving surgery (52.0%), axillary lymph node dissection (64.8%), and had received chemotherapy (70.3%), radiotherapy (72.5%), and endocrine therapy (76.6%).

**Table 1:** Patient demographics and clinical characteristics.

	Value
Age (years), mean (S.D.)	61.7 (7.8)
BMI (kg/m^2^), mean (S.D.)	26.0 (4.0)
**Breast surgery type, number (%)**	
BCS	142 (52.0)
Mastectomy	131 (48.0)
**Axillary surgery type, number (%)**	
SLNB	96 (35.2)
ALND	177 (64.8)
**Tumor histology type, number (%)**	
IDC	176 (64.5)
ILC	57 (20.9)
DCIS	3 (1.0)
Other	37 (13.6)
Chemotherapy (yes), number (%)	192 (70.3)
Radiotherapy (yes), number (%)	198 (72.5)
Endocrine therapy (yes), number (%)	209 (76.6)

S.D., standard deviation; IQR, interquartile range; BMI, body mass index; BCS, breast-conserving surgery; SLNB, sentinel lymph node biopsy; ALND, axillary lymph node dissection; IDC, intraductal carcinoma; ILC, intralobular carcinoma; DCIS, ductal carcinoma *in situ*.

Of the 273 patients, 22 (8.0%) used neuropathic pain medications, such as tricyclic antidepressants, gabapentinoids (gabapentin or pregabalin), or serotonin and norepinephrine reuptake inhibitors. Further, 63 (23.1%) used mild opioids, non-steroidal anti-inflammatory drugs, or paracetamol.

Descriptive statistics of the assessed pain-related and psychological instruments, including temperament and character dimensions and HrQoL, are presented in Table 2. Of the patients, 53.1% (145/273) reported moderate to severe pain (NRS ≥ 4/10) at any site of the body in BPI. The distribution of pain located at the previously operated area in 62.3% (170/273) and only in other site of the body in 37.7% (103/273) of the cases. Additionally, 18 patients presented evoked pain at previously operated area in clinical examination.

**Table 2:** Descriptive statistics of the assessed instruments.

	Value
**Short-Form 36, mean (S.D.)**	
Physical HrQoL	66.5 (21.6)
Mental HrQoL	70.7 (21.4)
**Temperament and Character Inventory, mean (S.D.)**	
Novelty seeking	17.3 (6.2)
Harm avoidance	14.5 (7.1)
Reward dependence	15.3 (4.0)
Persistence	3.7 (1.9)
Self-directedness	34.5 (5.8)
Cooperativeness	34.8 (5.1)
Self-transcendence	14.4 (6.6)
**Brief Pain Inventory, median (IQR)**	
**Operated area (the breast, the axilla, and the upper arm) (NRS 0–10)**	
Pain severity	1 (0–3)
Pain interference	1 (0–2)
**Other body locations (NRS 0–10)**	
Pain severity	3.5 (2–5)
Pain interference	2 (1–5)
**Total (all body locations) (NRS 0–10)**	
Pain severity	4 (2–6)
Pain interference	3 (1–5)
**Hospital Anxiety and Depression Scale, mean (S.D.)**	
Anxiety	5.0 (3.2)
Depressive symptoms	3.4 (3.4)
**Pain Catastrophizing Scale, median (IQR)**	
Pain catastrophizing	7 (1–3)
**Insomnia Severity Index, mean (S.D.)**	
Insomnia symptoms	8.1 (5.7)

S.D., standard deviation; IQR, interquartile range; HrQoL, health-related quality of life; NRS, Numerical Rating Scale. Missing values: Hospital Anxiety and Depression Scale, (anxiety), *n* = 1; Hospital Anxiety and Depression Scale (depression), *n* = 1; Insomnia Severity Index, *n* = 3; Pain Catastrophizing Scale, *n* = 6; Brief Pain Inventory (other body locations), *n* = 19.

Physical HrQoL correlated significantly with temperament dimensions NS (*p* = 0.016) and HA (*p* < 0.001), and with character dimensions SD (*p* = 0.013) and CO (*p* = 0.046). Mental HrQoL correlated significantly with HA (*p* < 0.001), SD (*p* < 0.001), and CO (*p* = 0.005) (Table 3). Of the potential mediator variables unsignificant correlations were found only for pain severity with HA (*p* = 0.385), SD (*p* = 0.607), and CO (*p* = 0.633), and for pain interference with SD (*p* = 0.303) and CO (*p* = 0.402). According to the criteria for mediation analysis, non-significant correlations were excluded from the mediation analyses. All of the potential mediator variables correlated significantly with dimensions of physical and mental HrQoL (Table A1). Intercorrelations between the potential mediator variables are presented in Table A2.

**Table 3:** Pearson correlation coefficients of the study variables.

	NS	HA	RD	*P*	SD	CO	ST
Physical HrQoL	0.153*	−0.268***	−0.001	−0.033	0.157*	0.126*	0.018
Mental HrQoL	0.035	−0.374***	0.045	−0.096	0.380***	0.179**	−0.013
Depressive symptoms	−0.113	0.446***	−0.095	0.140*	−0.468***	−0.191**	−0.041
Anxiety	−0.021	0.446***	0.067	0.180**	−0.395***	−0.187**	0.076
Symptoms of insomnia	0.007	0.261***	0.092	0.237***	−0.269***	−0.198**	0.077
Pain catastrophizing	−0.112	0.227***	0.018	0.044	−0.233***	−0.145*	0.029
Pain severity	0.049	0.058	−0.023	0.090	0.006	−0.057	0.001
Pain interference	−0.112	0.122*	0.029	0.125*	−0.031	−0.077	0.042

HrQoL, health-related quality of life; NS, novelty seeking; HA, harm avoidance; RD, reward dependence; P, persistence; SD, self-directedness; CO, cooperativeness; ST, self-transcendence. Bolded values indicate statistically significant result. For statistical significance: *p < 0.05; **p < 0.01; ***p < 0.001.

### Parallel mediation models

**Figure 2:**
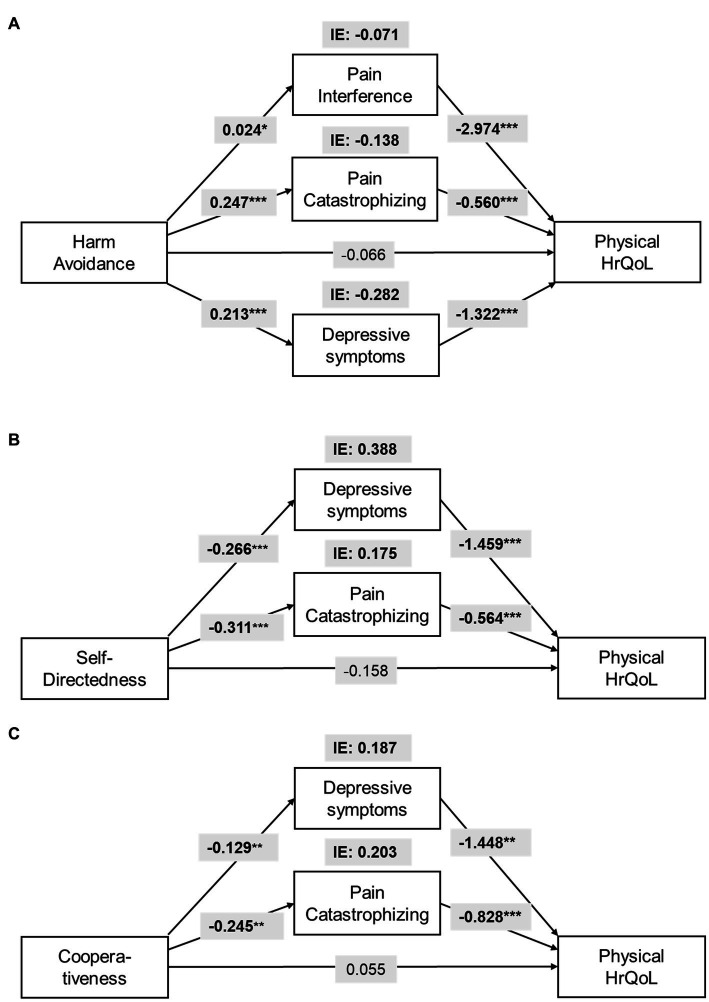
Parallel mediation model for the indirect effects (IE) of **(A)** *harm avoidance* (HA), **(B)** *self-directedness* (SD), and **(C)** *cooperativeness* (CO) on physical Health-related Quality of Life (HrQoL). All models are controlled for age and intensity of chronic pain. For statistical significance: *p < 0.05; **p < 0.01; ***p < 0.001.

**Figure 3:**
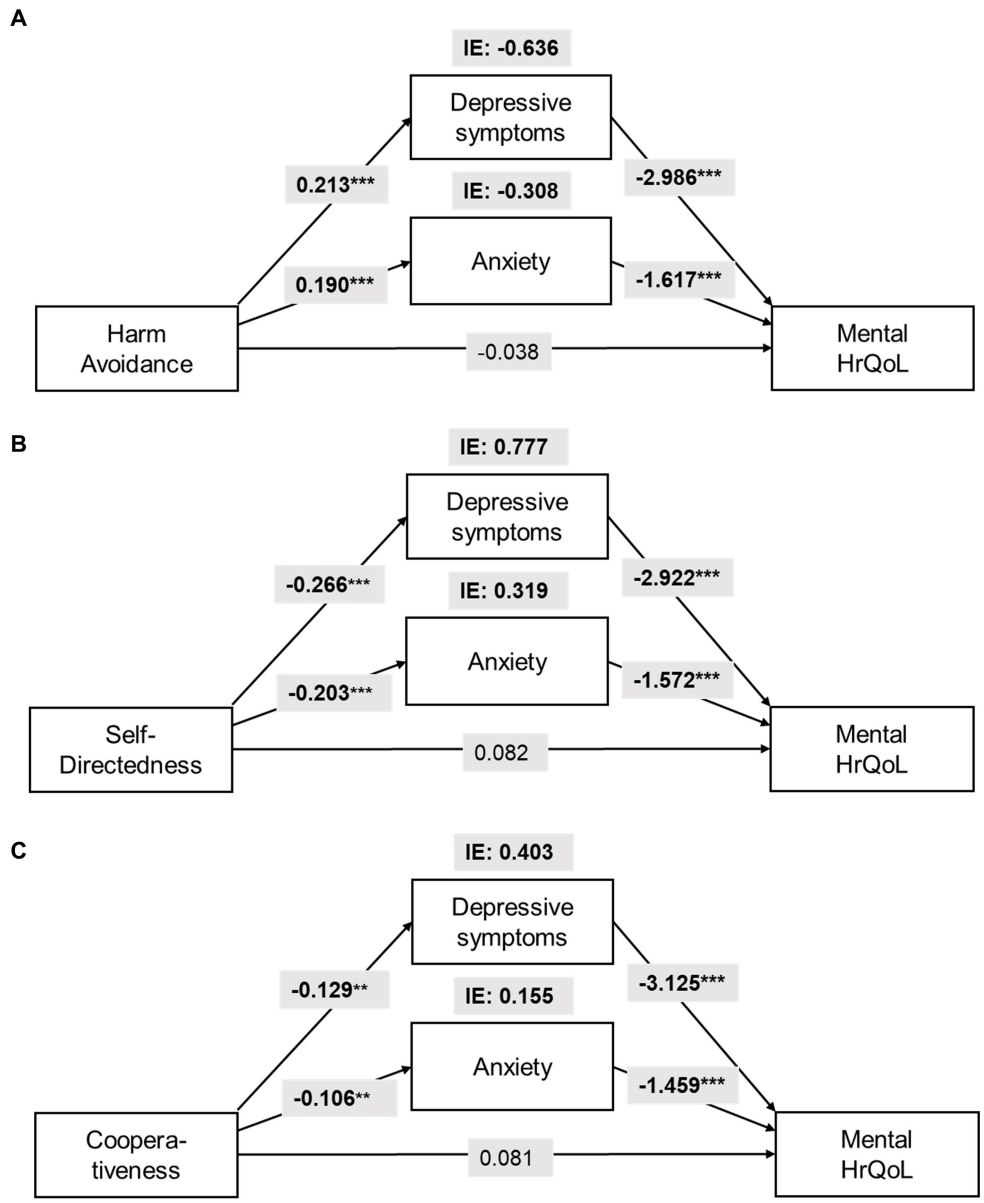
Parallel mediation model for the indirect effects (IE) of **(A)** *harm avoidance* (HA), **(B)** *self-directedness* (SD), and **(C)** *cooperativeness* (CO) on mental Health-related Quality of Life (HrQoL). All models are controlled for age and intensity of chronic pain. For statistical significance: **p < 0.01; ***p < 0.001.

The total effect of *harm avoidance* (HA) on physical HrQoL (β_total_ = −0.665, SE = 0.165, *p* < 0.001) and mental HrQoL (β_total_ = −1.071, SE = 0.177, *p* < 0.001) was significant, while the direct effects was not. Depressive symptoms (IE_depression_ = −0.282, CI_95%_: LL = −0.508 to UL = −0.109), pain catastrophizing (IE_catastrophizing_ = −0.138, CI_95%_: LL = −0.278 to UL = −0.044), and pain interference (IE_interference_ = −0.071, CI_95%_: LL = −0.171 to UL = −0.007), fully mediate the relationship between HA and physical HrQoL and contribute to the overall IE (Figure 2A). For mental HrQoL, depressive symptoms (IE_depression_ = −0.636, CI_95%_: LL = −0.924 to UL = −0.420) and anxiety (IE_anxiety_ = −0.308, CI_95%_: LL = −0.459 to UL = −0.146) fully mediate the effect of HA and contributed to the overall IE (Figure 3A).

The total effect of *self-directedness* (SD) on physical HrQoL (β_total_ = 0.609, SE = 0.187, *p* = 0.001) and mental HrQoL (β_total_ = 1.319, SE = 0.199, *p* < 0.001) was significant, while the direct effects was not. Depressive symptoms (IE_depression_ = 0.388, CI_95%_: LL = 0.173 to UL = 0.650), and pain catastrophizing (IE_catastrophizing_ = 0.175, CI_95%_: LL = 0.059 to UL = 0.343) fully mediate the relationship between SD and physical HrQoL (Figure 2B). For mental HrQoL, depressive symptoms (IE_depression_ = 0.777, CI_95%_: LL = 0.510 to UL = 1.091) and anxiety (IE_anxiety_ = 0.319, CI_95%_: LL = 0.152 to UL = 0.524) fully mediated the effect of SD (Figure 3B).

The total effect of *cooperativeness* (CO) on physical HrQoL (β_total_ = 0.584, SE = 0.264, *p* = 0.028) and mental HrQoL (β_total_ = 0.759, SE = 0.265, *p* = 0.005) was significant, while the direct effects was not. Depressive symptoms (IE_depression_ = 0.187, CI_95%_: LL = 0.050 to UL = 0.409), and pain catastrophizing (IE_catastrophizing_ = 0.203, CI_95%_: LL = 0.046 to UL = 0.429) fully mediate the relationship between CO and physical HrQoL (Figure 2C). For mental HrQoL, depressive symptoms (IE_depression_ = 0.403, CI_95%_: LL = 0.155 to UL = 0.745) and anxiety (IE_anxiety_ = 0.155, CI_95%_: LL = 0.040 to UL = 0.316) fully mediated the effect of CO (Figure 3C).

## Discussion

### Main findings

We showed that the psychological and pain-related variables convey the effects of temperament and character on HrQoL in BC survivors. The temperament dimension HA showed a negative effect on physical and mental HrQoL via mood, catastrophic thinking, and pain interference. Character dimensions SD and CO showed a protective effect against factors like mood and catastrophic thinking on physical and mental HrQoL. Mediators for temperament and character differed for physical and mental HrQoL.

### Personality characteristics and HrQoL

In previous studies, personality characteristics have consistently been associated with various, and especially psychosocial dimensions of HrQoL (Huang et al., 2017). Direct and indirect effects influence HrQoL via emotional reactivity, coping, and health behavior. A dynamic balance between temperament and character influence an individual’s psychosocial adaptation and well-being (Cloninger et al., 2010; Wong and Cloninger, 2010). Temperament refers to automatic and associative reactions, such as primary emotions and fear, whereas character, a collection of self-concepts learned during psychosocial development, refers to the interpretation of the significance of the internal stimuli, such as pain, or external events, such as cancer diagnosis (Cloninger et al., 2010).

In our study, pain-related factors, such as pain interference and pain catastrophizing, mediated the negative effect of HA on physical but not mental HrQoL. Likewise, pain catastrophizing mediated the effect of SD and CO only for physical HrQoL. Pain interference and pain catastrophizing are both strongly related especially with somatic sensations and interpretations of the state of physical health. Pain interference refers to the reactive dimension of pain and its interference with daily functions and HrQoL (Cleeland and Ryan, 1994). Pain catastrophizing refers to a negative cognitive-affective and coping responses to pain driving attention toward worsening somatic sensations and illness perception (Quartana et al., 2009). Catastrophic interpretations and hypervigilance toward somatic sensations facilitate the fear-avoidance behavior and negative emotions (Crombez et al., 2012), which are associated with HA temperament (Naylor et al., 2017). Persistent pain itself and pain-related catastrophizing may affect physical HrQoL, for example, by preventing the rehabilitation of upper limb dysfunction after BC surgery (De Groef et al., 2017). Additionally, in individuals with heightened tendency for pain-related catastrophic thinking, the poorer coping strategies (Quartana et al., 2009) may explain the shown association between SD, CO, and pain catastrophizing (Cloninger et al., 2010).

Lowered mood and symptoms of insomnia are crucial factors affecting HrQoL in BC survivors (Mokhatri-Hesari and Montazeri, 2020). We found that depressive symptoms mediated the effect of HA, SD, and CO on both physical and mental HrQoL. In other words, depressive symptoms indirectly convey the effects of these personality characteristics on HrQoL. High HA is a long-term risk factor for depressive symptoms and clinical depression (Cloninger et al., 2006), which are, beside of anxiety, clinically important comorbidities in BC survivors (Abrahams et al., 2018; Wang et al., 2018; Mokhatri-Hesari and Montazeri, 2020). On the other hand, low SD is a significant predictor for cognitive dysfunctions relating to clinical depression (Richter and Eisemann, 2002). Anxiety mediated the effect of HA, SD, and CO only on mental HrQoL, thus, forming an indirect link between temperament and character on psychosocial aspects of perceived health. We found no mediating effect of symptoms of insomnia on HrQoL, which may reflect a mediating effect of HA on depressive symptoms instead of multifactorial symptoms of insomnia (Lee et al., 2012).

Our results suggest that HA temperament is a vulnerability factor linked to mood, pain-related interference, and pain catastrophizing. In clinical pain, mood has been shown to have a significant mediative effect on HrQoL (Galvez-Sánchez et al., 2020). Our results suggest a protective role of SD and CO character dimensions on both physical and mental HrQoL. Character dimensions, like SD and CO, have been associated with various aspects of well-being (Cloninger et al., 2010) and they have been suggested to be protective factors for mood disorders (Richter and Eisemann, 2002; Cloninger et al., 2006). In BC survivors, social support and functioning are important predictors for HrQoL. The protective effect of SD and CO on HrQoL may reflect the differences in coping abilities, such as willpower, self-regulation, personality maturation, and social coping (Cloninger et al., 1993).

The full mediation between HA, SD, CO, and the dimensions of HrQoL might reflect the complex adaptive system of human personality as temperament and character refer to vulnerability or protective factors rather than deterministic factors for health and well-being. On the other hand, full mediation may be partly explained by statistical reasons as the non-significant direct effect in our data does not mean lack of a true direct effect in a population.

### Clinical implications

According to our results, high HA individuals may be vulnerable to lowered mood and pain-related disability and, therefore, clinically significant psychological burden. Bedside clinical evaluation of the tendencies for general worrying, fearfulness, or fatigability, related with high HA, may provide new insights to patient selection for clinical interventions. As personality characteristics effect long-term clinical well-being of BC survivors, interventions targeted to personality characteristics could improve clinical treatment outcomes (Durá-Ferrandis et al., 2017) and person-centered clinical practice (Wong and Cloninger, 2010). Moderate to strong evidence suggest a relationship of fatigue with anxiety and depression, persistent pain, and catastrophic thinking in BC survivors (Abrahams et al., 2018; Mokhatri-Hesari and Montazeri, 2020), which can be used as targets for psychological interventions for improving psychosocial health and HrQoL, such as cognitive-behavioral therapies (CBT) (Ye et al., 2018) and acceptance and commitment therapy interventions (ACT) (Hughes et al., 2017; González-Fernández and Fernández-Rodríguez, 2019).

Additionally, our results suggest a protective role for certain character dimensions (namely SD and CO). These might be a target for therapeutic interventions in patients with pain and high HA-related features (Wong and Cloninger, 2010). Cognitive behavioral therapies are an important method for empowering the vulnerable patient group with persistent pain and impaired HrQoL as well as BC survivors (Ye et al., 2018) as characters SD and CO have in previous studies presented as predictors for psychological well-being and social coping (Cloninger et al., 2010; Wong and Cloninger, 2010).

After BC treatments, persistent postsurgical pain (Wang et al., 2018; Mustonen et al., 2019) and upper limb dysfunctions (De Groef et al., 2017) have a negative effect on HrQoL. These possibly reflect mechanisms of catastrophic thinking and fear of pain on BC survivors at long term (De Groef et al., 2017), but also issues related to worry about BC in general and possible treatment side-effects (Mokhatri-Hesari and Montazeri, 2020). The clinical relevance of our findings may highlight the importance of effective pain management, psychoeducation, BC treatment methods, and other multidisciplinary therapeutical interventions (Wang et al., 2018).

### Limitations

There are limitations of the study. First, our data consisted only of women who had been treated for BC. Thus, the results cannot be directly generalized to healthy participants, other chronic diseases, or males. Additionally, as the patients with ongoing cancer treatments might suffer from acute side effects of the adjuvant therapies and pronounced psychosocial burden of disease, the results may not be generalized to the patients with active disease. However, a strength of this study is the homogenous and well characterized cohort of patients treated for BC.

Secondly, the limited sample size may limit making prominent conclusions. However, to the best of our knowledge, this is one of the largest patient sample related with temperament and character in BC survivors.

Thirdly, the study design was observational and cross-sectional. Therefore, despite of the mediation models, conclusions about the causality between studied variables cannot be drawn (Edwards et al., 2016). In the future, more complex structural models may be needed to study associations between different personality profiles and HrQoL. Longitudinal datasets are needed to confirm the causal associations of personality characteristics and potential mediators on dimensions of HrQoL.

Fourthly, most measurements were based on self-reports, which may differ from clinical assessment and therefore affect the results. It is possible that the measurement of personality dimensions can be affected by the presence of persistent pain (Fishbain et al., 2006).

## Conclusion

High *harm avoidance* temperament is a potential vulnerability factor for greater psychological burden of disease and impaired HrQoL in BC survivors. High *self-directedness* and *cooperativeness* character may protect from the negative effects of anxiety and depressive symptoms on HrQoL. From a multidisciplinary point of view, our results may provide new insights about the risk-and target-factors for clinical interventions and emphasize the importance of effective pain management to improve psychological health and HrQoL in BC survivors.

## Data availability statement

The original contributions presented in the study are included in the article/supplementary material, further inquiries can be directed to the corresponding author.

## Ethics statement

The studies involving human participants were reviewed and approved by Ethics Committee of the Helsinki and Uusimaa Hospital District (reference number: 149/13/03/00/14). The patients/participants provided their written informed consent to participate in this study.

## Author contributions

EK, HH, and RS designed the study. HH and RS collected the data. TA analyzed the data supervised by JL and prepared figures and tables. TA wrote the first draft of the manuscript. All authors contributed to the article and approved the submitted version.

## Funding

This study was funded by the European Union FP7 (# Health_F2-2013-602891), NeuroPain.

## Conflict of interest

EK has provided consultancy to Orion Pharma and Pfizer and HH to TEVA, Allergan, and Lilly, unrelated to this work.

The remaining authors declare that the research was conducted in the absence of any commercial or financial relationships that could be construed as a potential conflict of interest.

## Publisher’s note

All claims expressed in this article are solely those of the authors and do not necessarily represent those of their affiliated organizations, or those of the publisher, the editors and the reviewers. Any product that may be evaluated in this article, or claim that may be made by its manufacturer, is not guaranteed or endorsed by the publisher.

## Figures and Tables

**Table A1:** Intercorrelations between physical and mental Health-related Quality of Life (HrQoL) and potential mediator variables.

	1	2	3	4	5	6	7
1. Physical HrQoL (SF-36)							
2. Mental HrQoL (SF-36)	0.616***						
3. Depressive symptoms (HADS)	−0.428***	−0.712***					
4. Anxiety (HADS)	−0.377***	−0.639***	0.702***				
5. Symptoms of insomnia (ISI)	−0.352***	−0.409***	0.390***	0.406***			
6. Pain catastrophizing (PCS)	−0.497***	−0.398***	0.348***	0.365***	0.340***		
7. Pain severity (BPI)	−0.463***	−0.242***	0.164**	0.140**	0.273***	0.282***	
8. Pain interference (BPI)	−0.540***	−0.300***	0.202***	0.175**	0.285***	0.326***	0.848***

HrQoL, health-related quality of life; SF-36, Short-Form 36; HADS, Hospital Anxiety and Depression Scale; ISI, Insomnia Severity Index; PCS, Pain Catastrophizing Scale; BPI, Brief Pain Inventory. Bolded values indicate statistically significant result. For statistical significance: **p < 0.01; ***p < 0.001.

**Table A2:** Intercorrelations between temperament and character dimensions.

	1	2	3	4	5	6
1. Novelty seeking						
2. Harm avoidance	−0.415***					
3. Reward dependence	0.172**	0.001				
4. Persistence	0.001	0.032	0.098			
5. Self-directedness	0.043	−0.475***	0.135*	−0.133*		
6. Cooperativeness	0.119	−0.295***	−0.051	−0.051	0.461***	
7. Self-transcendence	0.225***	−0.185**	0.296**	0.296***	0.051	0.228***

Bolded values indicate statistically significant result. For statistical significance: *p < 0.05; **p < 0.01; ***p < 0.001.

## References

[ref1] Aalto A. Aro A. R. Teperi J. (1999). RAND-36 terveyteen liittyvän elämänlaadun mittarina. Mittarin luotettavuus ja suomalaiset väestöarvot. Stakes Tutkimuksia:101.

[ref2] Abrahams H. J. G. Gielissen M. F. M. Verhagen C. A. H. H. V. M. Knoop H. (2018). The relationship of fatigue in breast cancer survivors with quality of life and factors to address in psychological interventions: a systematic review. Clin. Psychol. Rev. 63, 1–11. doi: 10.1016/j.cpr.2018.05.004, PMID: 29852324

[ref3] Andersen K. G. Kehlet H. (2011). Persistent pain after breast cancer treatment: a critical review of risk factors and strategies for prevention. J. Pain 12, 725–746. doi: 10.1016/j.pain.2010.12.005, PMID: 21435953

[ref4] Bjelland I. Dahl A. A. Haug T. T. Neckelmann D. (2002). The validity of the hospital anxiety and depression scale. An updated literature review. J. Psychosom. Res. 52, 69–77. doi: 10.1016/S0022-3999(01)00296-3 11832252

[ref5] Bonacchi A. Miccinesi G. Guazzini M. Rossi A. Bacci S. Toccafondi A.. (2012). Temperament and character traits associated with health-related quality of life in cancer patients. Tumori J 98, 377–384. doi: 10.1177/030089161209800316, PMID: 22825515

[ref6] Caffo O. Amichetti M. Ferro A. Lucenti A. Valduga F. Galligioni E. (2003). Pain and quality of life after surgery for breast cancer. Breast Cancer Res. Treat. 80, 39–48. doi: 10.1023/A:1024435101619 12889597

[ref7] Cleeland C. S. Ryan K. M. (1994). Pain assessment: global use of the brief pain inventory. Ann. Acad. Med. Singap. 23, 129–138. PMID: 8080219

[ref8] Cloninger C. R. Svrakic D. M. Przybeck T. R. (1993). A psychobiological model of temperament and character. Arch. Gen. Psychiatry 50, 975–990. doi: 10.1001/archpsyc.1993.01820240059008 8250684

[ref9] Cloninger C. R. Svrakic D. M. Przybeck T. R. (2006). Can personality assessment predict future depression? A twelve-month follow-up of 631 subjects. J. Affect. Disord. 92, 35–44. doi: 10.1016/j.jad.2005.12.034, PMID: 16442638

[ref10] Cloninger C. R. Zohar A. H. Cloninger K. M. (2010). Promotion of well-being in person-centered mental health care. Focus 8, 165–179. doi: 10.1176/foc.8.2.foc165, PMID: 26146491 PMC4486313

[ref11] Conrad R. Wegener I. Geiser F. Kleiman A. (2013). Temperament, character, and personality disorders in chronic pain. Curr. Pain Headache Rep. 17:318. doi: 10.1007/s11916-012-0318-3, PMID: 23338770

[ref12] Crombez G. Eccleston C. Van Damme S. Vlaeyen J. W. Karoly P. (2012). Fear-avoidance model of chronic pain: the next generation. Clin. J. Pain 28, 475–483. doi: 10.1097/AJP.0b013e3182385392 22673479

[ref13] De Groef A. Meeus M. De Vrieze T. Vos L. Van Kampen M. Christiaens M.-R.. (2017). Pain characteristics as important contributing factors to upper limb dysfunctions in breast cancer survivors at long term. Musculoskelet. Sci. Pract. 29, 52–59. doi: 10.1016/j.msksp.2017.03.005, PMID: 28319882

[ref14] Durá-Ferrandis E. Mandelblatt J. S. Clapp J. Luta G. Faul L. A. Kimmick G.. (2017). Personality, coping, and social support as predictors of long-term quality-of-life trajectories in older breast cancer survivors: CALGB protocol 369901 (a lliance). Psychooncology 26, 1914–1921. doi: 10.1002/pon.4404, PMID: 28219113 PMC5563496

[ref15] Edwards R. R. Dworkin R. H. Sullivan M. D. Turk D. C. Wasan A. D. (2016). The role of psychosocial processes in the development and maintenance of chronic pain. J. Pain 17, T70–T92. doi: 10.1016/j.jpain.2016.01.001, PMID: 27586832 PMC5012303

[ref16] Ferlay J. Colombet M. Soerjomataram I. Parkin D. M. Piñeros M. Znaor A.. (2021). Cancer statistics for the year 2020: an overview. Int. J. Cancer 149, 778–789. doi: 10.1002/ijc.33588, PMID: 33818764

[ref17] Fishbain D. A. Cole B. Cutler R. B. Lewis J. Rosomoff H. L. Rosomoff R. S. (2006). Chronic pain and the measurement of personality: do states influence traits? Pain Med. 7, 509–529. doi: 10.1111/j.1526-4637.2006.00239.x 17112364

[ref18] Galvez-Sánchez C. M. Montoro C. I. Duschek S. Del Paso G. A. R. (2020). Depression and trait-anxiety mediate the influence of clinical pain on health-related quality of life in fibromyalgia. J. Affect. Disord. 265, 486–495. doi: 10.1016/j.jad.2020.01.129, PMID: 32090776

[ref19] Gerbershagen H. J. Rothaug J. Kalkman C. J. Meissner W. (2011). Determination of moderate-to-severe postoperative pain on the numeric rating scale: a cut-off point analysis applying four different methods. Br. J. Anaesth. 107, 619–626. doi: 10.1093/bja/aer195, PMID: 21724620

[ref20] González-Fernández S. Fernández-Rodríguez C. (2019). Acceptance and commitment therapy in cancer: review of applications and findings. Behav. Med. 45, 255–269. doi: 10.1080/08964289.2018.1452713, PMID: 29558259

[ref21] Hayes A.F.. Introduction to mediation, moderation, and conditional process analysis. A regression-based approach. New York, NY: The Guilford Press; (2013).

[ref22] Huang I. C. Lee J. L. Ketheeswaran P. Jones C. M. Revicki D. A. Wu A. W. (2017). Does personality affect health-related quality of life? A systematic review. PLoS ONE 12:e0173806. doi: 10.1371/journal.pone.0173806, PMID: 28355244 PMC5371329

[ref23] Hughes L. S. Clark J. Colclough J. A. Dale E. McMillan D. (2017). Acceptance and commitment therapy (ACT) for chronic pain. Clin. J. Pain 33, 552–568. doi: 10.1097/AJP.0000000000000425 27479642

[ref24] Kaunisto M. A. Jokela R. Tallgren M. Kambur O. Tikkanen E. Tasmuth T.. (2013). Pain in 1, 000 women treated for breast cancer: a prospective study of pain sensitivity and postoperative pain. Anesthesiology 119, 1410–1421. doi: 10.1097/ALN.0000000000000012, PMID: 24343286

[ref25] Laroche F. Perrot S. Medkour T. Cottu P. H. Pierga J. Y. Lotz J. P.. (2017). Quality of life and impact of pain in women treated with aromatase inhibitors for breast cancer. A multicenter cohort study. PLoS ONE 12:e0187165. doi: 10.1371/journal.pone.0187165, PMID: 29117210 PMC5678681

[ref26] Lee S. Kim S. J. Park J. E. Cho S. J. Cho I. H. Lee Y. J. (2012). Biogenetic temperament and character in insomnia and depression. J. Psychosom. Res. 72, 383–387. doi: 10.1016/j.jpsychores.2012.01.016, PMID: 22469281

[ref27] Meretoja T. Leidenius M. Tasmuth T. Sipilä R. Kalso E. (2014). Pain at 12 months after surgery for breast cancer. J. Am. Med. Assoc. 311, 90–92. doi: 10.1001/jama.2013.278795 24381969

[ref28] Miettunen J. Kantojärvi L. Ekelund J. Veijola J. Karvonen J. T. Peltonen L.. (2004). A large population cohort provides normative data for investigation of temperament. Acta Psychiatr. Scand. 110, 150–157. doi: 10.1111/j.1600-0047.2004.00344.x, PMID: 15233716

[ref29] Mokhatri-Hesari P. Montazeri A. (2020). Health-related quality of life in breast cancer patients: review of reviews from 2008 to 2018. Health Qual. Life Outcomes 18, 338–325. doi: 10.1186/s12955-020-01591-x, PMID: 33046106 PMC7552560

[ref30] Morin C. M. Belleville G. Belanger L. Ivers H. (2011). The insomnia severity index: psychometric indicators to detect insomnia cases and evaluate treatment response. Sleep 34, 601–608. doi: 10.1093/sleep/34.5.601, PMID: 21532953 PMC3079939

[ref31] Mustonen L. Aho T. Harno H. Sipilä R. Meretoja T. Kalso E. (2019). What makes surgical nerve injury painful? A 4-year to 9-year follow-up of patients with intercostobrachial nerve resection in women treated for breast cancer. Pain 160, 246–256. doi: 10.1097/j.pain.0000000000001398, PMID: 30234699 PMC6319585

[ref32] Naylor B. Boag S. Gustin S. M. (2017). New evidence for a pain personality? A critical review of the last 120 years of pain and personality. Scan J Pain 17, 58–67. doi: 10.1016/j.sjpain.2017.07.011, PMID: 28850375

[ref33] Preacher K. J. Hayes A. F. (2004). SPSS and SAS procedures for estimating indirect effects in simple mediation models. Behav. Res. Meth. Instrum. Comput. 36, 717–731. doi: 10.3758/BF03206553, PMID: 15641418

[ref34] Quartana P. J. Campbell C. M. Edwards R. R. (2009). Pain catastrophizing: a critical review. Expert. Rev. Neurother. 9, 745–758. doi: 10.1586/ern.09.34, PMID: 19402782 PMC2696024

[ref35] Richter J. Eisemann M. (2002). Self-directedness as a cognitive feature in depressive patients. Pers. Individ. Differ. 32, 1327–1337. doi: 10.1016/S0191-8869(01)00121-0

[ref36] Sullivan M. J. L. Bishop S. Pivik J. (1995). The pain catastrophizing scale: development and validation. Psychol. Assess. 7, 524–532. doi: 10.1037/1040-3590.7.4.524

[ref37] Treanor C. Donnelly M. (2015). A methodological review of the short form health survey 36 (SF-36) and its derivatives among breast cancer survivors. Qual. Life Res. 24, 339–362. doi: 10.1007/s11136-014-0785-6, PMID: 25139502

[ref38] Wang K. Yee C. Tam S. Drost L. Chan S. Zaki P.. (2018). Prevalence of pain in patients with breast cancer post-treatment: a systematic review. Breast 42, 113–127. doi: 10.1016/j.breast.2018.08.105, PMID: 30243159

[ref39] Wong K. M. Cloninger C. R. (2010). A person-centered approach to clinical practice. Focus 8, 199–215. doi: 10.1176/foc.8.2.foc199, PMID: 26029006 PMC4448138

[ref40] Ye M. Du K. Zhou J. Zhou Q. Shou M. Hu B.. (2018). A meta-analysis of the efficacy of cognitive behavior therapy on quality of life and psychological health of breast cancer survivors and patients. Psychooncology 27, 1695–1703. doi: 10.1002/pon.4687, PMID: 29500842

